# Seroprevalence of antibodies to astrovirus in chickens in Grenada, West Indies

**DOI:** 10.14202/vetworld.2017.636-639

**Published:** 2017-06-13

**Authors:** Ravindra Nath Sharma, Romane Dufayet, Thomas Maufras, Kathryn O’ Connell, Keshaw Tiwari

**Affiliations:** 1 Department of Pathobiology, School of Veterinary Medicine, St. George’s University, Grenada, West Indies; 2 Department of Preclinical Studies, Ecole Nationale Veterinaire De Toulouse, France (DVM students, on Internship Program at St. George’s University Grenada)

**Keywords:** astrovirus, chicken astroviruses, chickens, Grenada, seroprevalence

## Abstract

**Aim:**

Chicken astroviruses (CAstV) are known to cause mild gastroenteritis, growth depression, and even mortality in poultry, especially in chickens, turkeys, and ducks. To the best our knowledge, there is no published information on CAstV in Grenada. This study was conducted to determine the prevalence of astrovirus in chickens in Grenada.

**Materials and Methods:**

Blood samples from 366 indigenous chickens and 92 commercial chicken layers were collected from all parishes of the island and tested for antibodies against CAstV using commercial enzyme-linked immunosorbent assay.

**Results:**

The seroprevalence of antibodies against astrovirus was 57.6% (95%, Confidence interval [CI]: 47.4-67.2) in commercial layers and 61.5% (95%, CI: 56.4-66.3) in indigenous chickens. The results show the presence of infection throughout the island.

**Conclusion:**

The results show the infection with CAstV in approximately half of the chicken population in Grenada. This is the first report on the prevalence of CAstV in chickens in Grenada and the Caribbean region.

## Introduction

Astroviruses are small, round, non-enveloped, single-stranded, positive-sense RNA viruses, typically 28-30 nm in diameter [1]. The name astrovirus comes from the Greek “astron,” meaning star, describing the 5-6 pointed star-like surface projections detected by negative-stained electron microscopy [2]. Astroviruses belong to the Astroviridae family, which is divided into two genera: *Mamastrovirus* (mammalian astroviruses) and *Avastrovirus* (avian astroviruses) [1].

*Mamastroviruses* are associated with gastroenteritis outbreaks in infants [2,3] and elderly people [4] and are an important cause of gastroenteritis in immunocompromised individuals [5]. Astroviruses also cause severe disease, including diarrhea and gastroenteritis in several mammals and birds, such as swine, sheep, cats, dogs, cattle, turkeys, guinea fowls, and ducks [6].

*Mamastrovirus* infection was not reported from avian species, yet. However, recently, Pancovics *et al*. [7] detected a mammalian-like astrovirus in bird, European roller (*Coracias garrulous*) in Hungary.

To date, five different astroviruses have been identified in avian species [8]. In turkeys, two types of astroviurus (TAstV) have been identified, TAstV-1 and TAstV-2. Both types of TAstVs are genetically and immunologically distinct [9]. Astroviruses cause severe outbreaks of gastroenteritis in turkeys [9,10]. In ducks, the virus is called Duck AstroVirus, primarily known as a picornavirus, Duck Hepatitis Virus Type II and this virus causes fatal hepatitis in young ducklings [11].

In chickens, there are two species of astroviruses, which are antigenically and genetically distinct. Avian nephritis virus (ANV) was originally classified as a picornavirus but later as an astrovirus on the basis of nucleotide sequence [12]. ANV affects young chicks and causes growth depression and nephritis with swollen kidneys, prominent ureters, and visceral gout. There are necrosis and degeneration of the epithelial cells of the proximal convoluted tubules with interstitial infiltration of granulocytes and lymphocytes in the kidneys. They also have caused outbreaks of gout in broilers in India [13]. Recently, Espinoza *et al*. [14] identified a new strain of ANV in Brazilian commercial chicken flocks. De Wit *et al*. [15] reported a new group of ANV in chicken and turkeys with enteric and locomotion disorders. Zhao *et al*. [16] found 89% ANV and 4% chicken astroviruses (CAstV) in pigeons (*Columbia livia*) in China, during an outbreak of diarrhea.

A second species of astovirus, CAstV, was isolated from broiler chicks. CAstV is associated with poor weight gain and enteric disease in chickens [17]. Recently, Todd *et al*. [18] identified two genetically different isolates of CAstV, named CAstV 612 and CAstV 11672, who share the low levels of antigenic relatedness.

Worldwide, isolated published information is available on the seroprevalence of avian astroviruses. To the best of our knowledge, in the Caribbean region, including Grenada, there is no information on CAstV. In the present study, the seroprevalence of antibodies for astrovirus (CAst-V) in indigenous and commercial chickens of Grenada is reported for the first time.

## Materials and Methods

### Ethical approval

The project was approved by Institutional Animal care and Use Committee (IACUC # 12005).

### Study area

Grenada is the southernmost island country in the southeastern Caribbean Sea. The island is divided into six parishes. Blood from 366 randomly selected indigenous chickens between the ages of 1-2 years and 92 blood samples collected randomly from layers in the age group between 1 and 1.5 years from commercial flocks from all six parishes were tested for antibodies against CAstV. Around 30% of the Grenadian households keep indigenous chickens for their family use. The flock of indigenous chickens varies from 10 to 50 chickens per household. Blood from indigenous chickens was collected from five birds in flocks consisting of up to 10 birds and from 20% of birds in flocks with a size >10 birds. From commercial layers, blood from around 15 chickens from 2 to 3 farms in each parish was collected.

### Sample preparation and detection of antibodies

A volume of 2-3 ml blood sample was collected from the basilic vein (wing vein) of the chickens. Sera were separated by centrifugation of blood at 3000 g for 10 min and frozen at −80°C until tested by enzyme-linked immunosorbent assay (ELISA). Sera were tested by ELISA. The CAstV Group B antibody test kit from Biocheck (UK) Ltd. 11, Millfarm Business Park, Millfield Road, Hounslow, London (Manufacturer) was used, according to manufacturer’s protocol.

### Statistical analysis

Confidence interval (CI) and Fisher’s exact test were calculated using the following website: (http://www.mccallumlayton.co.UK/states/CI Calcproportion.aspx).

## Results

The seroprevalence of CAstV in indigenous chickens and commercial layer chickens is presented in Table-1.

**Table-1:** Seroprevalence of antibodies for CAstV in chickens in Grenada, West Indies.

Type of chicken	Numbers of samples	Positive	% Positive (CI)
Commercial layer chickens	92	53	57.6 (47.4-67.2)
Indigenous chickens	366	225	61.5 (56.4-66.3)

CAstV=Chicken astroviruses

Out of 366 indigenous chickens tested, 225 were positive for antibodies to CAstV (61.5% CI: 95%: 56.4-66.3%). Out of 92 tested commercial layer chickens, 53 were positive for antibodies to CAstV, (57.6% CI: 95%: 47.4-67.2%).

There was no significant difference in seropositivity of antibody for CAstV in commercial layer chicken and indigenous chicken (p=0.5508) (Fisher’s exact test).

## Discussion

The presence of antibodies to CAstV has been demonstrated in chickens by previous researchers in many countries of the world. Baxendale and Mebatsion [17] and Todd *et al*. [18] showed widespread CAstV antibodies among broiler, parent, grandparent, and great-grandparent flocks in the United Kingdom, Europe, Australia, and the United States. Oluwayelu and Todd [19] reported serological evidence of antigenically and genetically distinct CAstV (strains CAstV 612 and CAstV 11672) in Nigerian indigenous chickens.

The prevalence of antibodies to CAstV varied in chicken flocks in various countries. Todd *et al*. [18] reported 53% seroprevalence in parent flock, 28% in grandparent, and 21% in great grandparent chicken flocks in the UK. Seropositivity was demonstrated for CastV 612 and CAstV 11672 strains. Oluwayelu and Todd [19] found 4.0% seropositivity for CAstV 612% and 8.0% for CAstV 11672 in Nigerian indigenous chickens. Smyth *et al*. [20] detected CAstV in gut content of 96% of chickens from four broiler flocks in the UK. In our study, we found 57.6% of commercial chickens and 61.5% of indigenous chickens positive for CAstV antibodies. Although in our research using ELISA, it was not possible to test chickens for specific CAstV strains, the results suggest the wide circulation of CAstV in both commercial and indigenous chickens in Grenada. Since a vaccine for CAstV is not available, the chickens in Grenada are not vaccinated. This indicates natural exposure of chickens to CAstV. Moreover, since the seropositive birds were from all the parishes of Grenada, a widespread infection is indicated in the country.

Various clinical signs associated with CAstV included mainly enteritis and growth depression [10,15,17]. Sajewicz-Krukowska *et al*. [21] recently reported “white chick” condition associated with CAstV infection. CAstV not only caused embryo/chick mortality but also weakness and white plumage of hatched chicks. Other researchers, however, found no correlation between the presence or absence of antibodies for CAstV and signs of growth depression [17,18,22]. All chickens tested under this research looked healthy without any clinical signs of growth retardation and enteritis. To prove the association between infection and clinical disease, more detailed study is needed.

Astroviruses are very resistant in the environment and resist inactivation by most routinely used disinfectants [23-25]. According to Schultz-Cherry *et al*. [25], the only products completely effective at inactivation were 0.3% formaldehyde, 1.5% Virkon S, 0.1% b-propiolactone, and 90% methanol. CAstVs are also resistant to temperature (up to 60°C for 10 min) and to low pH [24]. These findings show that when a flock is infected with CAstV, it is difficult to completely rid the flock of infection. Grenadian poultry farmers need to be educated on the application of biosecurity to prevent CAstV infection.

### Validation of results with real time PCR

Initially the research was planned to perform seroprevalence of astrovirus, hence we only collected blood and completed serology. Finding high sero positivity for astrovirus in chickens, we were prompted to conduct molecular techniques. For PCR we needed fecal material from chickens which was not collected in this project. Extra funding was granted by the University and fecal swabs specimens are being collected. We hope to complete PCR on indigenous and commercial chickens by mid 2018. As far as authors know no research on astrovirus is going on in the Caribbean region.

## Conclusion

There is no published information on CAstV in Grenada and other Caribbean nations. A serological investigation to find out the presence of antibodies to CAstV in indigenous and commercial chickens was conducted in Grenada. The presence of antibodies in approximately half of the tested chickens, by use of ELISA, indicates a widespread infection with CAstV. Because of the lack of a vaccine, the need to educate poultry farmers in Grenada regarding the use of biosecurity to prevent CAstV has been emphasized.

This is the first report on the presence of CAstV antibodies in chickens in Grenada and the Caribbean nations.

## Author’s Contribution

RNS planning and oversee of the research project and manuscript writing; RD, TM, and KO’C helping KT in the collection of blood and performing the ELISA, KT collection of blood, performing ELISA, and review of the manuscript.
